# Delayed vacuolation in mammalian cells caused by hypotonicity and ion loss

**DOI:** 10.1038/s41598-024-79815-z

**Published:** 2024-11-26

**Authors:** Emily Zook, Yingzhou Edward Pan, Anna Wipplinger, Hubert H. Kerschbaum, Robert J. Clements, Markus Ritter, Tobias Stauber, Michael A. Model

**Affiliations:** 1 Department of Biological Sciences, Kent State University, Kent, OH USA; 2 Institute for Molecular Medicine, MSH Medical School Hamburg, Hamburg, Germany; 3 Center for Physiology, Pathophysiology and Biophysics, Institute of Physiology and Pathophysiology, Paracelsus Medical University, Salzburg, Austria; 4 Department of Biosciences and Medical Biology, University of Salzburg, Salzburg, Austria

**Keywords:** Hypotonicity, Lysosome, Vacuole, WNK kinase, Biochemistry, Cell biology

## Abstract

Prolonged exposure of mammalian cells to hypotonic environments stimulates the development of sometimes large and numerous vacuoles of unknown origin. Here, we investigate the nature and formation of these vacuoles, which we term LateVacs. Vacuolation starts after osmotic cell swelling has subsided and continues for many hours thereafter. Most of the vacuoles are positive for the lysosomal marker LAMP-1 but not for the autophagosomal marker LC3. Vacuoles do not appear to have acidic pH, as they exclude LysoTracker and acridine orange; inhibiting the V-ATPase with bafilomycin A1 has no effect on their formation. No LateVacs were formed in cells with a knockout of the essential LRRC8A subunit of the volume-regulated anion channel (VRAC). Since the main feature of cells recovered from hypotonic swelling should be reduced chloride concentration, we tested if chloride depletion can act as a signal for vacuolation. Indeed, four different low-chloride buffers resulted in the development of similar vacuoles. Moreover, vacuolation was suppressed in WNK1/WNK3 double knockouts or by the inhibition of WNK kinase, which is activated by low chloride; in hypotonic media, the WNK inhibitor had a similar effect. However, exposing cells to a low-sodium, high-potassium medium also resulted in vacuoles, which were insensitive to WNK. We conclude that vacuole development can be triggered either by the loss of chloride or by the loss of sodium.

## Introduction

Watery vacuoles are common in plants, yeast, and protists. They are conveniently observed under a positive phase contrast microscope as bright inclusions on a darker background due to a lower refractive index. Prominent vacuoles are not a standard attribute of cells of higher organisms but are known to develop in various, usually abnormal, conditions^1–6^. In most cases, the direct cause of water influx into vesicles remains unknown. The best understood vacuolation mechanism is the one operating in acidic lysosomes when they accumulate weak bases^7,8^. Once a neutral lipophilic molecule crosses the lysosomal membrane, it becomes protonated and trapped inside the organelle. The accumulation of charged molecules draws water into lysosomes, causing their expansion into large vacuoles.

Methuosis is another condition resulting in vacuoles. It is caused by the failure of macropinocytic vesicles to shrink and merge with endosomes and lysosomes, ensuring their persistence and gradual expansion^9,10^. Because of their pinocytic origin, their watery appearance is natural.

Vacuoles are also observed in a type of caspase-independent cell death known as paraptosis^11^. In paraptotic cells, vacuoles develop from the endoplasmic reticulum (ER), but the mechanism of water accumulation is unclear, since the proposed equivalent exchange of potassium for sodium^12^ is not expected to change their water content without engaging additional processes.

Vacuoles are a common marker of autophagy^13,14^. Autophagic vesicles start with a membrane, possibly of the ER origin, wrapping around protein aggregates or organelles destined for elimination. At a later stage, they fuse with lysosomes, after which hydrolytic enzymes reduce macromolecules to monomers, and membrane transporters return them to the cytosol. At some point, autophagic vacuoles become visible under phase contrast, which is a sign that their water content has increased. The relationship between the stage of autophagic digestion and water accumulation has not been studied extensively, but one may hypothesize that water-rich autophagosomes occur during the stage of macromolecular degradation (D. Klionsky, personal communication). Indeed, the refractive index depends on the mass/volume concentration of organic matter. During the early stages of digestion, the organic mass remains conserved, but the volume is expected to increase due to the accumulation of low-molecular weight osmolytes^15^.

Another signal leading to the enlargement of late endosomal/lysosomal vesicles is inhibition or non-functionality of PIKfyve, a lipid kinase converting PtdIns-3-P to PtdIns-3,5-P_2_ in the endocytic pathway, which is responsible for vesicle shrinkage along the endocytic route by activating vesicular TRPML and TPC channels^16–19^. Furthermore, PtdIns-3,5-P_2_ inhibits the lysosomal Cl^-^/H^+^ exchanger ClC-7 whose hyperactivation upon PIKfyve inhibition leads to a dramatic enlargement of lysosomes^20–23^. Notably, PIKfyve is an osmosensitive enzyme, which is activated by hyperosmolarity and eventually inhibited by hypoosmotic shock *in vivo*^6,24,25^.

Inhibition of p38 MAP kinases induces vacuolation in about half of tested mammalian cell lines^26^ but, interestingly, not in 3T3 fibroblasts, which was one of the cell lines investigated in this work. Like most other previously characterized vacuoles, those induced by p38 MAP kinase inhibition could be prevented with bafilomycin A1, an inhibitor of the lysosomal proton pump V-ATPase. Recently, it was reported that the latter type of vacuolation is due to combined inhibition of PIKfyve and p38 MAPKs^6^. Furthermore, deficiency of ATP13A2/PARK9 impairs lysosomal degradation and leads to an enlargement of lysosomes^27^.

In this work, we further studied the vacuoles that we had previously observed in mammalian cells incubated for many hours in strongly hypotonic solutions^28^. Those vacuoles did not accumulate dextran (therefore, were likely not of pinocytic origin), excluded calcein AM, carboxyfluorescein succinimidyl ester (CFSE), and sodium fluorescein. Depolymerization of the actin cytoskeleton by cytochalasin D enhanced vacuolation. The rest of the cytoplasm had normal protein concentration of about 0.2 g/ml, so that all the extra water was confined to the vacuoles. At that time, we failed to stain the vacuolar membranes and hypothesized that the watery compartments could represent second-phase inclusions, similar to necrotic blebs, outside of which the cytoplasm density remains unchanged^29^. In fact, vacuoles without visible membranes are known^4^, and the idea of liquid–liquid phase separation has been proposed as early as 1984^30^. This time, after testing other lipid stains, vacuolar membranes were detected.

To our knowledge, the first report of very rapid (within 5 min) vacuolation in the rabbit proximal tubule exposed to a hypotonic solution was published thirty years ago^31^. Only a slight osmotic gradient was required (270 mOsm/kg in the lumen vs. 290 mOsm/kg in the bath), while the presence of albumin or dextran in the bath solution prevented the formation of vacuoles. Ten years later, Iwasa et al. observed vacuoles in 3T3-L1 fibroblasts that started forming 30 min after a hypotonic shock^32^. The authors concluded that they originate from ER or Golgi, although not every vacuole could be stained with specific organelle markers. The vacuoles were reversible upon returning to normal osmolarity, which is common for mammalian vacuoles^4^. A more recent paper^33^ described large vacuoles in other cell types developing from various organelles within 10–30 min of hypotonic treatment. Another group also observed spherical organelles rapidly forming after hypotonic shock; whether or not they represent vacuoles is unclear because their water content has not been assessed^34^.

These published results have been interpreted in the sense that organelles may serve as water reservoirs during osmotic swelling. However, in our case, as well as in the study by Iwasa et al.^32^, the vacuoles continued to steadily grow for hours after the completion of the regulatory volume decrease (RVD), which takes only about 30 min^35^. This suggests that such slowly developing vacuoles respond not to water influx but to a different stimulus. In this paper, we will be referring to the vacuoles that form while the cells are still swollen (i.e. during RVD) as EarlyVacs and to the post-swelling vacuoles (i.e. after RVD) as LateVacs. The main result of this work is that LateVacs are stimulated by chloride or sodium depletion; we also present some evidence that they represent enlarged and dysfunctional lysosomes.

## Results

### Time course of vacuole formation

**Fig. 1:**
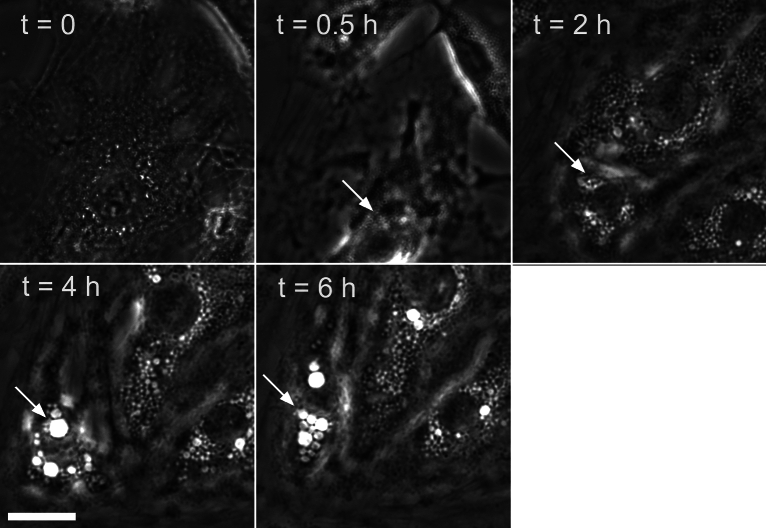
Phase contrast images showing the gradual development of vacuoles in 3T3 cells incubated in 33% DMEM. Approximately the same area was imaged at a 40 × magnification every time. The vacuoles were barely visible at 30 min; their substantial expansion occurred by 2 h and they became large and numerous by 4–6 h. Arrows point at representative vacuoles or their precursors (at 0.5 h). Based on 6 experiments with 3T3 cells and 102 cells analyzed, 92 ± 7% of the cells had at least one vacuole (V1), 86 ± 15% had at least five vacuoles (V5), and 74 ± 20% had at least one vacuole with a diameter of > 2 µm (V2µm). Scale bar, 25 μm.

Under a relatively low-resolution 40×/0.6 phase contrast objective, vacuoles in 3T3 became visible after 30 min of hypotonic treatment, although some roughness that marks their future location was discernible even before the treatment. The vacuoles began to noticeably enlarge after two or more hours (depending on the extracellular osmolarity) (Fig. 1), and were very prominent by the next day (e.g., Figs. 5, 6; see also Ref.^28^). Vacuoles in 3T3 cells usually developed sooner than in HeLa cells, and were more numerous. Restoration of isotonic medium led to gradual disappearance of vacuoles (Supplementary Fig. S1). In C28/I2 chondrocytes, LateVacs developed early in hypotonic medium and could be observed for at least one day afterwards (Supplementary Videos S1 and S2).

No vacuoles could be seen while the cells remained swollen. Conditions leading to persistent swelling, such as the prevention of RVD in HeLa cells with antimycin or ouabain, abolished vacuolation. At the same time, RVD in 3T3 cells was resistant to ouabain, and numerous vacuoles developed in practically every cell exposed to dilute medium (Supplementary Fig. S2). Neither did mutated 3T3 cells with impaired RVD (CRISPR-Cas9-depleted of LRRC8A^36^, and hence lacking functional volume-regulated anion channel (VRAC))^37,38^ develop any noticeable vacuoles (Supplementary Fig. S3). Similar results were obtained in VRAC-deficient *LRRC8A*^-/-^ C28/I2 cells (Supplementary Fig. S4, Supplementary Videos S3 and S4).

### The role of chloride depletion

After volume recovery in a hypotonic solution (RVD) is completed, cell volume, water content and the level of macromolecular crowding must be similar to the initial levels. Since RVD is predominantly achieved by a coupled exit of Cl^-^ and K^+^^39,40^, the most obvious difference between the initial and post-RVD cells must be the concentration of these ions. Of these two, chloride depletion is more likely involved in vacuolation because its intracellular concentration is lower, and therefore, the relative loss of chloride greatly exceeds the relative loss of potassium. In agreement with this hypothesis, the chloride channel blocker NPPB completely prevented hypotonicity-induced vacuoles in 3T3 cells (Supplementary Fig. S5).

**Fig. 2:**
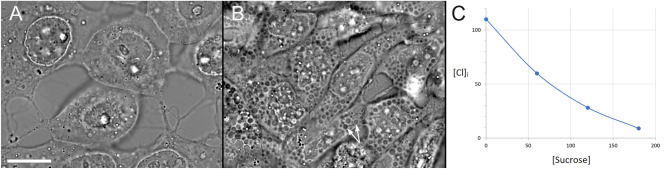
(**A**,**B**) Bright-field images of HeLa cells incubated for 6 h with VNGO in the low-sodium buffer (**A**) or in the same buffer, where 0.075 mM KCl was replaced with 0.15 M sucrose (**B**). Cell swelling and the absence of vacuoles were observed in the buffer with normal chloride content; however, partial replacement of chloride with sucrose reversed the swelling and resulted in multiple vacuoles. The high-sodium, low-potassium buffer had a similar effect. The V values were V1 = 80–89%, V5 = 74–89%, V2μm = 14–50% for sodium and potassium-based buffers supplemented with sucrose but zero in the absence of sucrose. Scale bar, 15 μm. (**C**) The simulation of the effect of sucrose at a high ion permeability due to ionophores (p = 1 for all ions, using the definitions adopted in the software) and with the Na,K pump inhibited by ouabain (β = 0.001) showed that at a steady state, intracellular chloride becomes depleted when part of external chloride is replaced with isotonic sucrose.

The initial indirect evidence for the role of chloride in the formation of LateVacs was obtained in an experiment with ionophores, in which HeLa cells were exposed to a combination of ionophores valinomycin, nigericin, gramicidin, and the Na^+^,K^+^ ATPase inhibitor ouabain (VNGO). Such a treatment stabilizes intracellular concentrations of cations at a near-extracellular level, while keeping cells viable for many hours^41^. When VNGO were applied in an isotonic buffer (whether high- or low-sodium), cells became and remained swollen without noticeable vacuoles. However, a partial replacement of chloride with isotonic sucrose reversed cell swelling and produced numerous vacuoles in all cells (Fig. 2). At a given osmolarity, cell volume at steady state correlates with chloride concentration^42^. Mathematical modeling using the program created by Alexey and Igor Vereninov (https://vereninov.com/cellionfluxes/)^43^ confirmed that the main effect of sucrose is a dramatic loss of intracellular chloride (Fig. 2C).

**Fig. 3:**
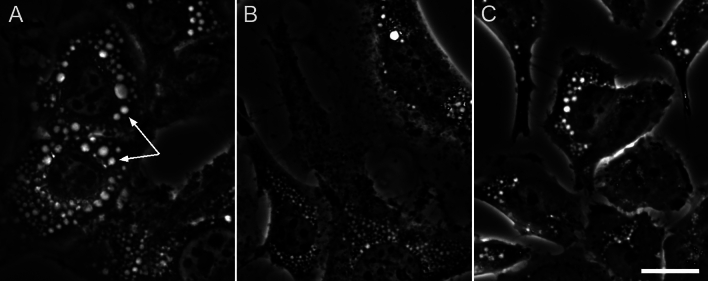
Phase contrast images of the LateVacs. (**A**) 3T3 cells incubated in the gluconate buffer. (**B**) 3T3 cells incubated in the low-sodium buffer. (**C**) HeLa cells incubated in the nitrate buffer. All the incubations were carried out for 6 h. These images cannot necessarily be seen as representative, because the size and the number of vacuoles varied between experiments (see Table 1), but they were common enough. Scale bar, 20 μm.

To directly test for a role of chloride in the generation of the LateVacs, we used chloride substitution for a different anion in the medium, which is a standard method to deplete intracellular chloride^44–47^. Thus, 3T3 cells were exposed to four different isotonic low-chloride buffers for 6 h. Like hypotonicity, all low-chloride solutions produced vacuoles (Fig. 3A, Table 1). An increase in vacuolation over control was observed in every low-chloride buffer by every parameter, although the differences were statistically highly significant (p < 0.005) only in methyl sulfate, nitrate and gluconate buffers (Fig. 3A), but not in the aspartate buffer. HeLa cells also developed vacuoles in low-chloride buffers (Fig. 3C). Unexpectedly, vacuoles also occurred in the low-sodium buffer with normal chloride content (Fig. 3B).

**Table 1:** Vacuolation in low-chloride buffers compared to the high-sodium buffer with normal chloride content.

Buffer	Experiments/Total cells	V1	V5	V2µm
High-NaCl (control)	4/205	48 ± 5	24 ± 6	6 ± 4
Nitrate	5/299	85 ± 3**	73 ± 6**	35 ± 17*
Methyl sulfate	5/238	82 ± 11**	64 ± 9**	29 ± 15*
Aspartate	4/180	67 ± 11*	30 ± 10	15 ± 10
Gluconate	4/200	66 ± 11	46 ± 12*	35 ± 11**

3T3 cells were exposed to the listed buffers for 6 h at 37 °C and analyzed by phase contrast imaging. The averages and standard deviations are shown. V1, V5, percent of cells with ≥ 1 and ≥ 5 clearly visible vacuole(s), respectively; V2µm, the percent of cells containing at least one vacuole with a diameter of > 2 µm. Asterisks indicate statistical significance: * for 0.005 < p < 0.05, and ** for p < 0.005, by unpaired two-tailed t-test.

**Fig. 4:**
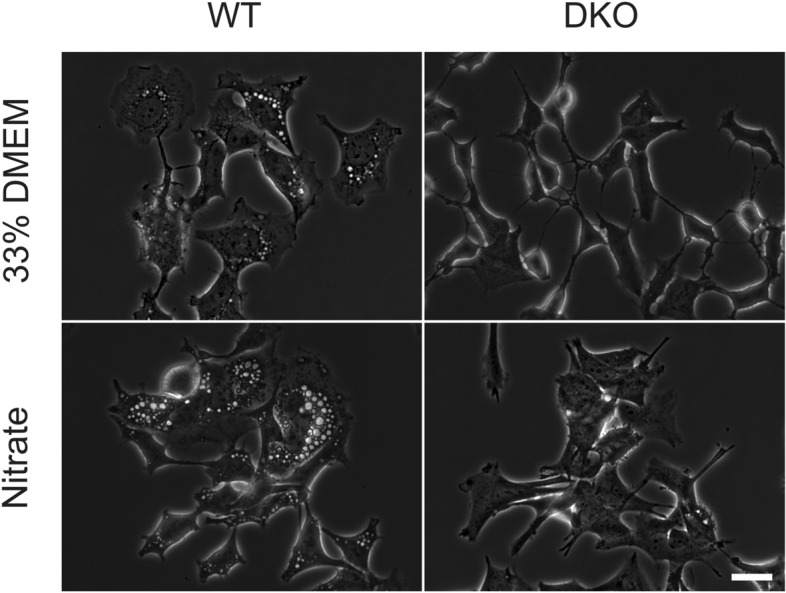
The effect of double WNK1/WNK3 knockout (DKO) on vacuolation. Wildtype and mutant HEK cells were observed after a 6 h incubation in the hypotonic solution (33% DMEM) or in a low-chloride buffer based on sodium nitrate. Also see Table 2 for quantification. Scale bar, 25 µm.

To confirm the specific role of chloride, we repeated the experiments in the presence of a pan-WNK-kinase inhibitor, WNK463, as well as with a WNK1/WNK3 knockout cell line^48^. The reason for investigating the role of WNK (“with no lysine” kinase) is that its various isoforms are inactivated by chloride and are believed to be involved in chloride sensing^49–51^. The results were consistent in that functional WNK is essential for the vacuolation response. Cells with suppressed WNK activity, either by genetic or pharmacologic means, had strongly diminished vacuolation in the low-chloride nitrate buffer and in 30% DMEM (Fig. 4, Table 2). At the same time, vacuolation in the low-sodium buffer was unaffected, pointing to the existence of different mechanisms.

**Table 2:** The effect of WNK inhibition (with 10 mM WNK463) or WNK kinase absence (DKO) on vacuolation. The conditions were as in Table 1.

Buffer	Treatment	Experiments/Total cells	V1	V5	V2µm
3T3					
Sodium nitrate	No inhibitor	5/247	83 ± 19	70 ± 20	56 ± 22
	+ WNK463	4/356	20 ± 12**	11 ± 11**	23 ± 20*
	+ DMSO	1/19	95	84	79
30% DMEM	No inhibitor	3/143	89 ± 19	73 ± 25	50 ± 35
	+ WNK463	3/143	40 ± 10*	28 ± 2	12 ± 9
Low-sodium	No inhibitor	3/176	80 ± 20	70 ± 22	47 ± 6
	+ WNK463	3/144	93 ± 3	79 ± 22	52 ± 20
HEK					
30% DMEM	WT	3/400	38 ± 8	23 ± 9	14 ± 3
	DKO	3/401	5 ± 2*	2 ± 1*	1 ± 1*
Sodium nitrate	WT	2/310	52 ± 0	39 ± 5	22 ± 4
	DKO	3/621	10 ± 3**	1 ± 2*	2 ± 2*

### Further characterization of the LateVacs

**Fig. 5:**
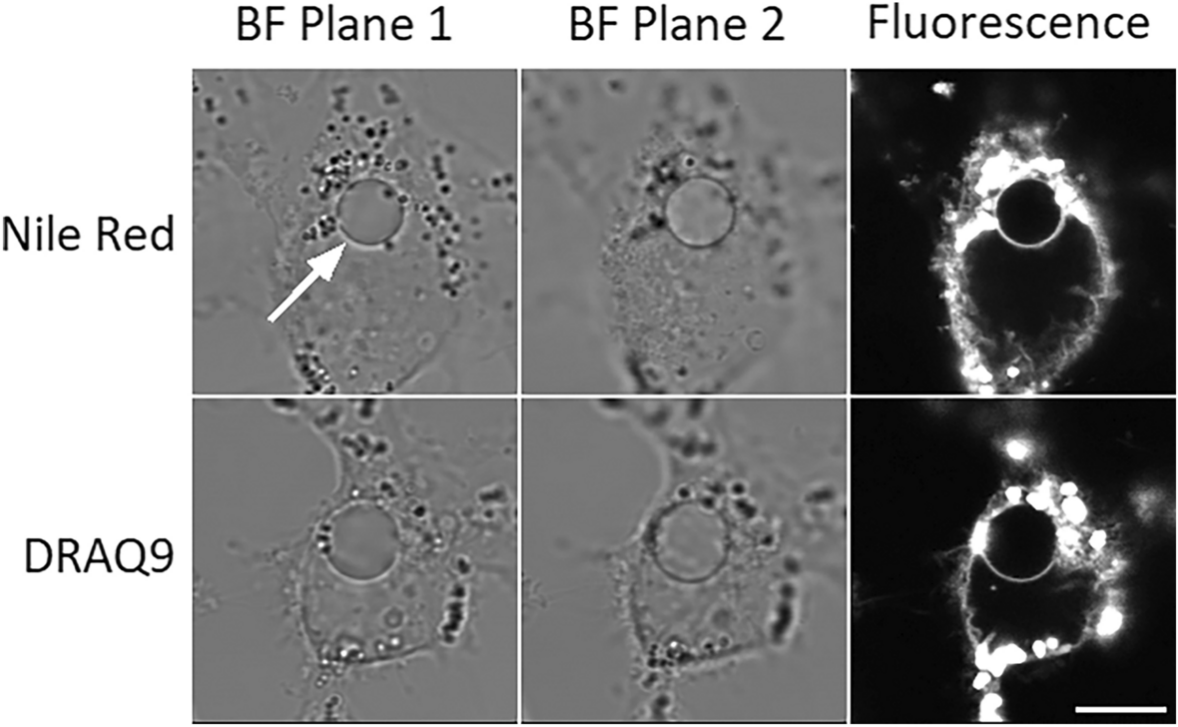
Confocal images of lipid staining reveal the membrane surrounding a single hypotonically-induced LateVac in HeLa (incubated overnight in 25% DMEM). The contrast inversion in bright field at defocusing indicates that the vacuoles have a low dry mass concentration: while optically denser compartments show a dark rim at overfocusing (the objective positioned too far from the object, BF1) and a light rim at underfocusing (objective brought too close, BF2), the vacuoles display the opposite behavior. Scale bar, 10 μm.

Our previous attempts to stain the membranes of the LateVacs with DiOC_18_(3) were unsuccessful^28^. This time, we tried the lipid stains Nile Red and DRAQ9; they both stained the periphery of the vacuoles, confirming the presence of a lipid membrane boundary (Fig. 5).

As in the previous publication, when we were unable to stain vacuoles with fluorescent markers^28^, none of the chemicals we tested additionally (acridine orange, sodium fluorescein, calcein-AM, and BCECF-AM) loaded into the vacuoles (Supplementary Fig. S6). The exclusion of acridine orange may indicate either alkalinization^52,53^ or poor membrane permeability. The membrane potential indicator DiBAC_4_(3) may have entered the vacuoles, but it also formed a rim around membranes; its fluorescence pattern is difficult to interpret (Supplementary Fig. S7).

### The origin of the LateVacs

**Fig. 6:**
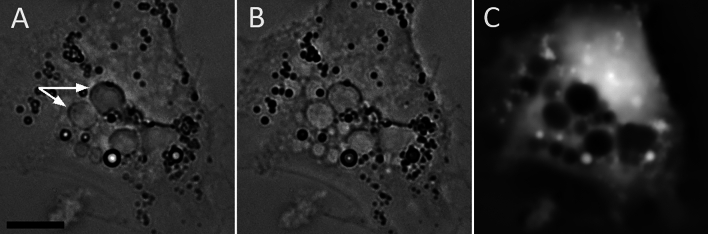
Vacuoles have a lower protein concentration than the cytosol. HeLa cells were incubated for 24 h in 35% DMEM. Two defocused brightfield images (**A**,**B**) and a TIE image computed from them (**C**).

Transport-of-intensity equation (TIE) images confirm that the intravacuolar protein concentration (Fig. 6A–C) is less than that in the cytosol (the latter was not measured every time but was estimated at 0.21 g/ml)^28^.

Because previous authors came to different conclusions regarding the origin of hypotonic vacuoles^32,33^, we conducted more experiments to shed additional light on this question. We first tested if the LateVacs could be autophagosomes. However, immunostaining for the autophagosomal marker LC3 failed to demonstrate any colocalization (Supplementary Fig. S8).

**Fig. 7:**
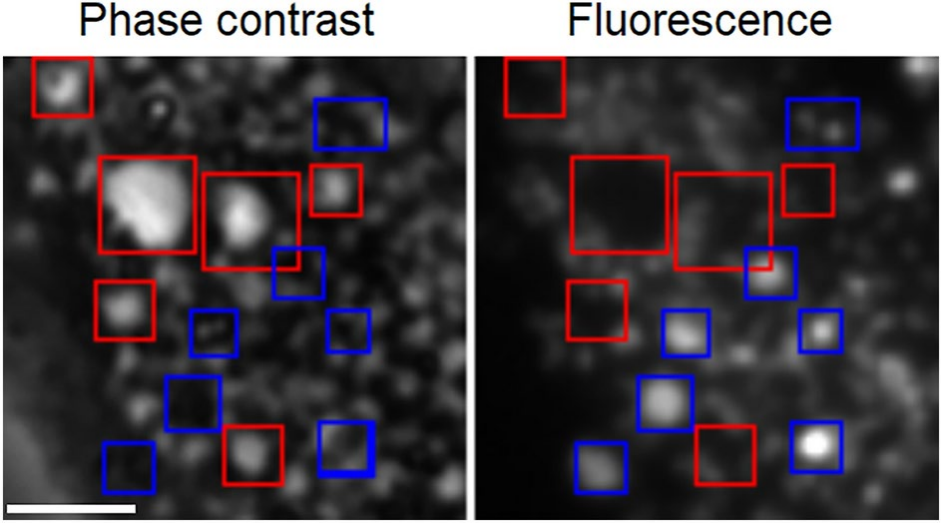
Negative correlation between phase contrast intensity and LysoTracker fluorescence. Red squares mark the vesicles with low protein density (i.e., vacuoles) and no fluorescence; blue squares mark the areas with high protein density and LysoTracker staining. Both phase contrast and fluorescence images of 3T3 cells were collected by the same 40 × phase contrast objective. Scale bar, 5 μm.

Lysosomes appeared to be another likely candidate as a progenitor of the vacuoles. Lysosomes are characterized by low pH (which forms the basis for their identification by the fluorescent probe LysoTracker) and high protein concentration^54,55^. When applied to 3T3 cells, lysotracker typically (though not always) colocalized with darker areas in phase contrast images, i.e., having a higher protein density, and that would indeed be expected for normal lysosomes. At the same time, watery vacuoles excluded the dye (Fig. 7).

**Fig. 8:**
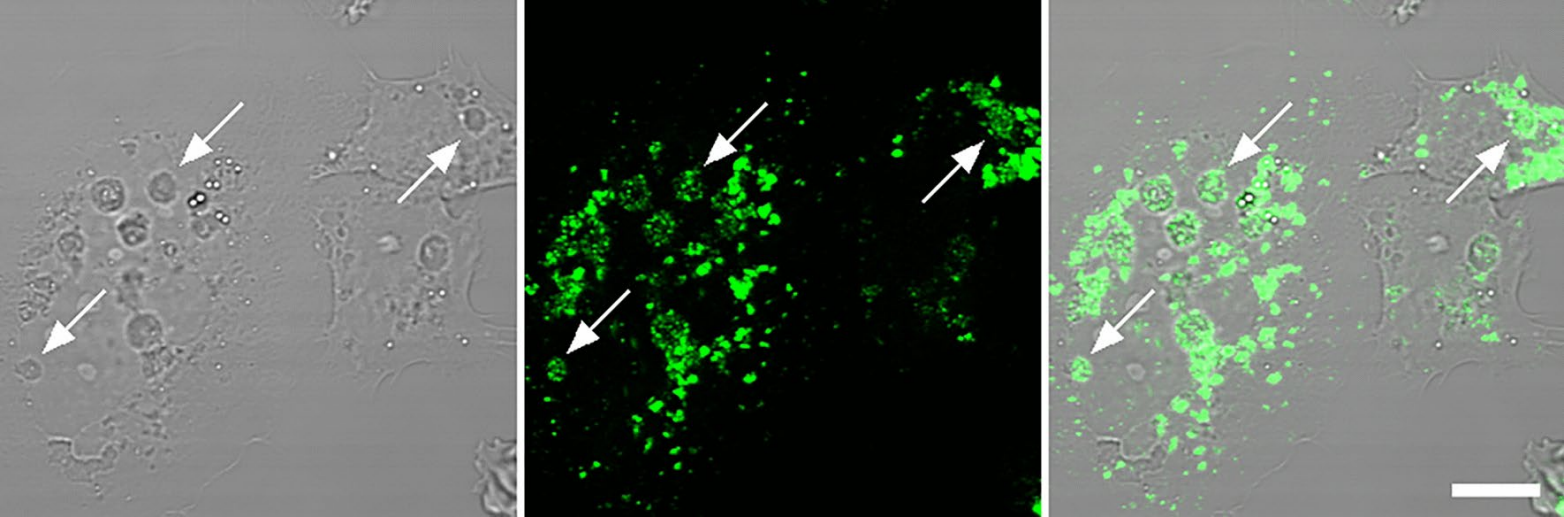
LAMP-1 staining of HeLa. Arrows point to some of the compartments that morphologically resemble vacuoles. All of them show the presence of LAMP-1. Scale bar, 10 µm.

Since live-cell staining by LysoTracker did not reveal the vacuole identity, we tested if their membranes express the lysosomal protein LAMP-1. Immunostaining of HeLa clearly showed the presence of the antigen inside every morphologically identifiable vesicle (Fig. 8). Although LAMP-1 is a membrane protein, it is possible that deformation or flattening of vacuoles during sample processing did not permit sufficient axial resolution to confirm its membrane localization. Lower-resolution images also confirmed the presence of LAMP-1 around vacuoles in 3T3 cells (Supplementary Fig. S9).

**Fig. 9:**
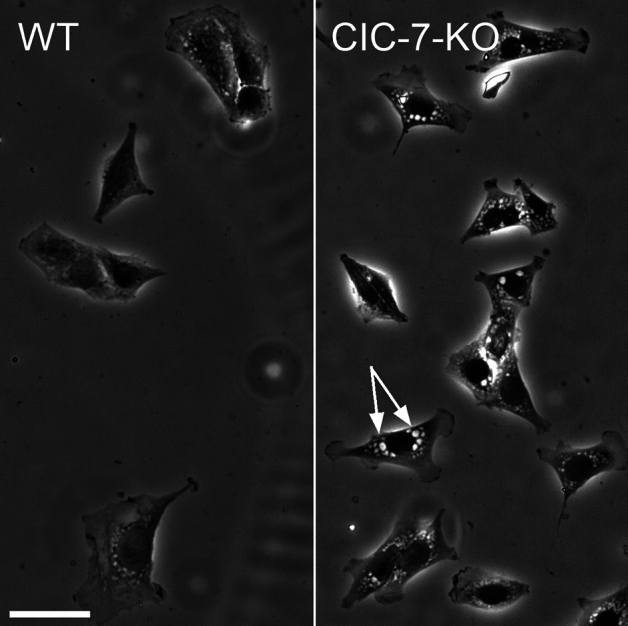
ClC-7 is dispensable for LateVac formation. Phase contrast images of mouse adult fibroblasts from wildtype (WT) or ClC-7-deficient *Clcn7*^-/-^ (ClC-7-KO) mice revealed prominent vacuoles after 6 h in hypotonic media. Neither cell type developed vacuoles in an isotonic medium Scale bar, 50 µm.

**Fig. 10:**
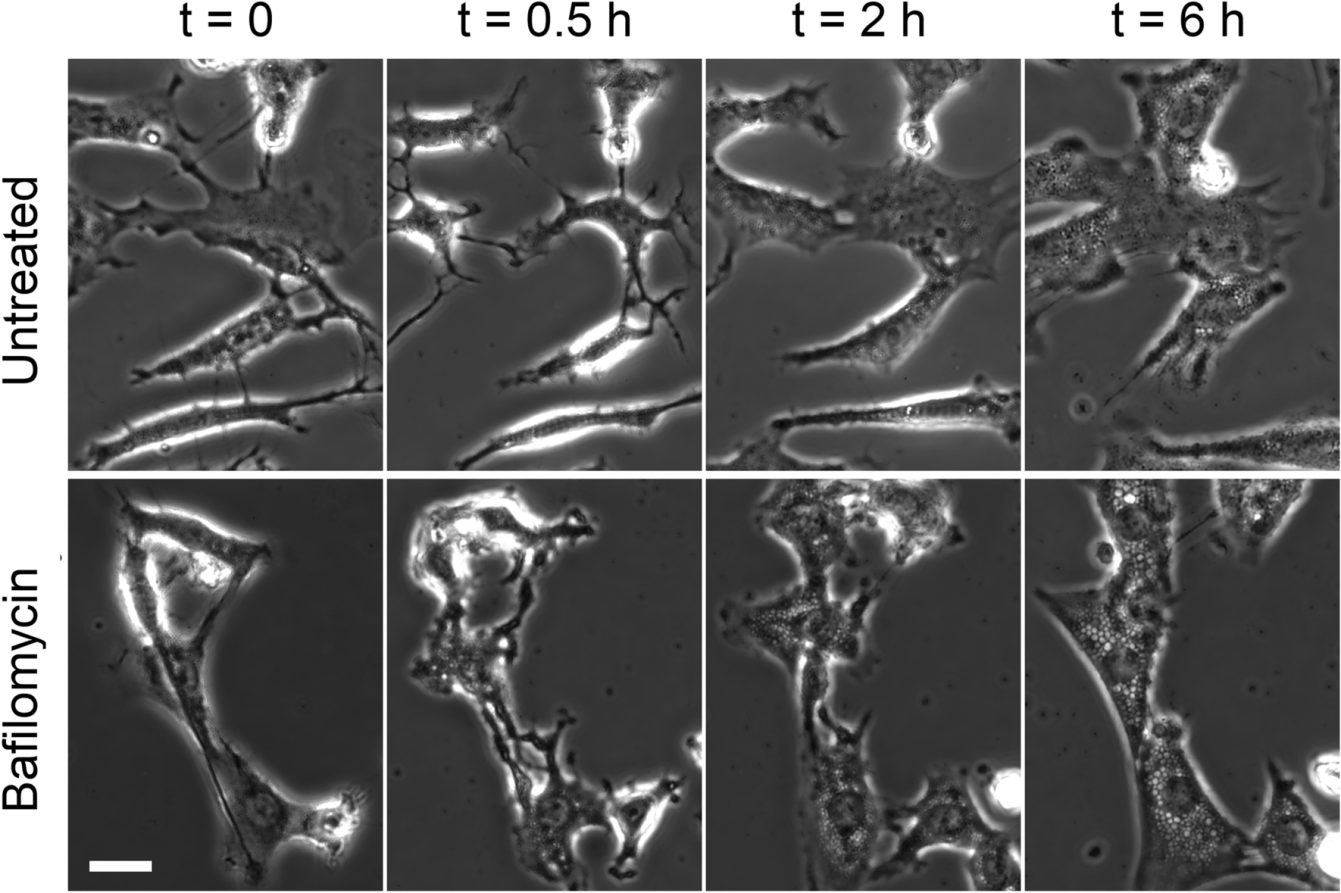
3T3 cells were exposed to 33% DMEM with or without bafilomycin A1 and observed under phase contrast at specified times. The numbers of visible vacuoles with and without bafilomycin A1 were as follows: 23 and 3 (0.5 h), 118 and 74 (2 h), and 227 and 77 (6 h), respectively. Scale bar, 25 μm.

Since at least a large fraction of the LateVacs were of lysosomal origin, we tested for the involvement of the chloride/proton exchanger ClC-7 in their formation. ClC-7 mediates pH gradient-driven accumulation of Cl^-^ into the lumen of lysosomes^56,57^, and a gain-of-function mutation of ClC-7 causes the formation of large intracellular vacuoles of lysosomal origin^20–22,58,59^. However, *Clcn7*^-/-^ mouse fibroblasts lacking ClC-7^60,61^ developed LateVacs in hypotonic media to a similar extent as did wildtype fibroblasts (Fig. 9). This result suggests that H^+^-mediated secondary active transport of Cl^-^ into the lysosomal lumen is not required for LateVac formation. This seems logical since the vacuoles do not appear to be acidic (see above). Moreover, the V-ATPase inhibitor bafilomyin A had no inhibitory effect on vacuoles but may have even slightly stimulated their formation (Fig. 10).

## Discussion

Vacuoles develop in mammalian cells in a perplexing variety of circumstances, such as starvation, disruption of signaling, phagocytosis, exposure to drugs, bacteria, viruses, cold, ischemia, hyper- and hypotonicity^3–5,62–65^. Here we have investigated a type of vacuoles initially observed in hypotonic solutions. This little-known phenomenon has been previously noted in cultured 3T3-L1 cells^32^ and subsequently reported in two other publications^33,34^.

Four cell types were used in this study: mouse fibroblasts, human cervical cancer HeLa cells, human embryonic kidney HEK cells, and human chondrocyte C28/I2 cells. Vacuoles in 3T3, HEK, and C28/I2 cells develop within a few hours and can be numerous, occupying a large portion of the cytoplasm (e.g., Supplementary Fig. S10 and Video S2). HeLa cells often require an overnight incubation in a hypotonic solution; their vacuoles are fewer and they are found in a smaller percentage of cells; but at the same time, they can be very large, such as in Fig. 5. Because of the ease of collecting statistics, the majority of experiments were eventually conducted on 3T3 cells. However, the main qualitative features of vacuoles—the lack of permeability for fluorescent dyes and dependence on chloride – are similar in HeLa and 3T3 cells.

Table 3 compares some features of hypotonic vacuoles established by the other authors^32,33^ and in our work. Clearly, there are major differences between the observations of Li et al.^33^ and ours. Moreover, the EarlyVacs were barely noticeable in 3T3 after 30 min (Fig. 1) and not visible at all in HeLa after 30 min in 50% DMEM (Supplemantary Fig. S11)—the conditions used by Li et al.^33^. The vacuoles described by Iwasa et al.^32^ more resemble ours, albeit with one major exception. Their vacuoles depended on chloride, whereas in our experiments, they resulted from the lack of chloride. We find it difficult to explain such discrepancies.

**Table 3:** Comparison of the features of hypotonic vacuoles in different studies.

Feature	Iwasa et al.^32^	Li et al.^33^	This work and Hollembeak and Model^28^
Cell types	3T3-L1	Cos, HeLa, Hap	3T3, HeLa, chondrocytes
Timing	> > 30 min	30 min	> > 30 min
Reversibility	Yes		Yes
Cl^-^ depletion	Inhibits		Stimulates
Cl^-^ channel blocker	Inhibits	Inhibits	Inhibits
Hg^2+^	No effect	Inhibits	No effect*
Bafilomycin A1		Inhibits	No effect
Cytochalasin			Stimulates
LAMP-1		+	+
Acridine orange	−		−
LysoTracker		+	−
Calcein AM		+	−
Dextran	+ / −	+	−

*Tentative conclusion based on insufficient data.

While some types of intracellular vacuoles have no detectable membrane^4^, we have confirmed that the LateVacs are membrane-bound organelles. This conclusion, however, relies on the assumption that our previous failure to stain vacuolar membranes with DiO^28^ was artifactual, whereas the positive results with DRAQ9 and Nile Red are real. A possible explanation of this difference is that DiO is more hydrophobic than the other two dyes, and if the vesicles are isolated from other organelles, DiO would have no easy access to them.

Antibody staining indicates that at least some of the LateVacs develop from lysosomes or result from fusion with them. The lack of staining by LysoTracker is likely due to the dissipation of the acidic pH. The latter is also suggested by the absence of staining with acridine orange, which is specific for acidic compartments. If the vacuoles represent enlarged lysosomes that have lost their acidity, the lack of inhibition of vacuole formation by bafilomycin A1 becomes understandable.

A separate question is why acetoxymethyl esters (such as calcein-AM, calcium green-AM, or BCECF-AM) do not accumulate in the vacuoles. One possible factor here is that hydrolysis renders the dyes hydrophilic before they enter the organelle; however, since de-esterification often takes tens of minutes, this explanation is less satisfactory. The more likely reason is that the LateVacs simply do not contain active esterases.

Our experiments with low-chloride solutions strongly suggest that WNK kinase activation induced by RVD-related chloride depletion is the main cause of delayed vacuolation. (Table 1). The role of chloride is also supported by the following observations: (a) inhibition of chloride-sensing WNK kinase suppresses vacuolation (Table 2); (b) WNK1/WNK3 double knockout cells are likewise resistant to vacuolation; (c) swollen cells that are expected to have high chloride concentration (since chloride is a major determinant of the cell volume^42^) do not form vacuoles (Supplementary Fig. S2); (d) the results with ionophores (Fig. 2). However, this picture is slightly muddled by the fact that vacuoles also developed in a low-sodium medium (Fig. 3). Further studies are needed to determine if sodium loss is somehow linked to the loss of chloride (although this does not follow from the cell model adopted in www.vereninov.com)^43^ or acts as an alternative stimulus for vacuolation.

Surprisingly little is known about the physiological mechanisms by which many types of mammalian vacuoles are formed. The majority of studies focused on molecular signaling rather than on the immediate causes of water accumulation^66^. In many cases, water influx and the dilution of organelles have only been assumed but not demonstrated directly. Here we would like to propose three possibilities.

1. Active uptake of ions or other osmolytes from the cytosol. The main vacuolar pump is the V-ATPase, but in our case its inhibitor bafilomycin A1 had no effect. One may hypothesize nevertheless that the V-ATPase remains active but inaccessible to bafilomycin A1. The binding site for bafilomycin A1 is located in the membrane-embedded domain^67^, and since the vacuolar membrane excludes many other compounds, it may exclude bafilomycin A1 as well.
2. Large-scale protein damage under highly abnormal conditions, such as hypotonicity or chloride deprivation. It can be mediated, for example, by free radicals and elevated calcium^68–70^. Damaged proteins are removed to lysosomes. The splitting of large molecules into smaller ones increases luminal osmolarity^15^. It would require active hydrolases and inactive exporters of the proteolytic fragments (catabolites). Catabolite transport may require sufficient luminal acidity^15^, and thus organic osmolytes may become trapped. The problem with this mechanism is that the vacuoles continue to expand for a long time, even after the lysosomes most likely became dysfunctional. Their growth by fusion alone cannot account for the large volume occupied by vacuoles in some cells.
  This hypothesis is similar to the hypothesis of “internal RVD”^33,71^, i.e., osmotically-driven removal of cytosolic water into vacuoles instead of the extracellular space. However, since LateVacs start developing at a time when cell volume is fully restored after osmotic swelling, the driving forces for water transport must be different.
3. Another factor to consider is the direct effect of alkalinization. The average isoelectric point of lysosomal proteins is 6.5; therefore, they carry a positive charge at normal lysosomal pH 4.7^72^ but become negative if lysosomes lose their acidity. Negative proteins will attract potassium from the cytosol (where its concentration is several times higher than in the vesicle). For a protein density of 300 mg/ml, the negative charge associated with proteins can be estimated at 45 mM^73^, so an import of 0.1 M potassium seems possible. This scenario is difficult to assess because of insufficient knowledge of lysosomal transporters and the lack of a comprehensive model that would incorporate processes both within the lysosomal and plasma membranes.

Thus, a fully satisfactory model of vacuole growth remains elusive. The involvement of WNK poses more interesting questions. Within the framework of the second model, WNK activated by hypotonicity^74^ or chloride depletion may (a) protect proteins from damage and denaturation, (b) interfere with their transport to lysosomes, (c) enable the release of digested products. Experimental evidence in support of any of these hypothetical roles of WNK is scant^75^. We have also tested for the possible involvement of the Na^+^-K^+^ pump, the hypothesis being that Na^+^ (and its counterion Cl^−^) are forced into vesicles, causing their swelling. However, the results with ouabain in 3T3 cells were negative overall, even if inconsistent (not shown); as for HeLa cells, they remained swollen in dilute solutions when pretreated with ouabain (Supplementary Fig. S2). In view of the subsequent results, the main argument against the role of the Na^+^-K^+^ pump is active vacuolation in low-sodium and low-chloride media.

## Conclusion

We have added another type of vacuole to the list of known mammalian vacuoles. These vacuoles slowly develop under hypotonic conditions, are stimulated by chloride depletion and require WNK activation as well as LRRC8A expression. Sodium depletion can also act as a signal for vacuole formation. At least some of these vacuoles seem to represent enlarged dysfunctional lysosomes.

## Methods

### Cell treatment

HeLa, 3T3 (both from ATCC, Manassas, VA, USA) and mouse adult fibroblasts (MAFs) were grown in DMEM (such as Corning, 343 mosm/kg, with 10% fetal bovine serum and antibiotics) at 37 °C and 5% CO_2_. Human immortalized C28/I2 cells were cultured in Dulbecco’s modified Eagle’s medium (DMEM)/HAM’s F‐12 medium (Biochrom, Darmstadt, Germany) supplemented with 5% fetal bovine serum (FBS Superior; Biochrom) and antibiotic‐antimycotic solution (100 U/ml penicillin, 0.1 mg/ml streptomycin, 0.25 μg/ml amphotericin‐B; Sigma‐Aldrich) at 37 °C and 5% CO_2_ (PMID: 7989586). LRRC8A knock-out was performed by CRISPR/Cas9-mediated deletion via introducing a frameshift insertion in exon 1. Next-generation sequencing showed a 75% knock-out efficiency in all alleles in the bulk population of C28/I2 cells. Single clones were isolated from the bulk population and the lack of LRRC8A expression on the protein level was confirmed by Western blot using polyclonal antibodies against LRRC8A (Cell Signaling Technology, catalog no. #24979) and histone H3 (Cell Signaling Technology, catalog no. #9715). LRRC8A-deficient 3T3 cells with a deletion of 2 and 5 nucleotides, respectively, in the open reading frames of the *Lrrc8a* alleles have been described previously^36^. Immortalized wildtype and ClC-7-deficient *Clcn7*^−/−^ MAFs^61^ were a gift from Thomas J. Jentsch (Leibniz-Forschungsinstitut für Molekulare Pharmakologie (FMP), Berlin, Germany). Wildtype and WNK1/WNK3 double-knockout HEK cells^48^ were a gift from Arohan Subramanya (University of Pittsburgh). Hypotonic media were prepared by diluting DMEM with deionized water. Low-chloride buffers were prepared with 10 mM Hepes, 5.5 mM glucose, 1 mM MgCl_2_, 1.25 mM CaCl_2_, 5% FBS, and one of the following: (a) 120 mM methyl sulfate, (b) 120 mM sodium nitrate with 30 mM sucrose (for isoosmolarity), (c) 120 mM L-aspartic acid, (d) 150 mM sodium gluconate with 60 mM sucrose, (e) 150 mM NaCl (high-sodium buffer), or (f) 150 mM KCl (low-sodium buffer, where FBS was the only source of sodium). The osmolarities of all buffers were between 290 mmol/L and 300 mmol/L. Chloride concentrations in the low-chloride buffers were not measured but were estimated to be approximately 9.5 mM. When using Hepes-based buffers, cells were kept at 37 °C in normal atmosphere. Additional treatments included 0.1–0.2 mM bafilomycin A1 (up to 5 mM in one experiment; InvivoGen, San Diego, CA), 0.5 mM ouabain (Sigma-Aldrich, St. Louis, MO, USA), 15 μM antimycin, 0.5 mM of 5-Nitro-2-(3-phenylpropylamino)benzoic acid (NPPB, Cayman Chemical, Ann Arbor, MI, USA) diluted from a 0.2 M stock in ethanol, 10 mM WNK463 (Focus Biomolecules, Plymouth Meeting, PA, USA). The drugs were added at the beginning of incubation. To equilibrate intracellular and extracellular potassium and sodium, we used either low-sodium or low-potassium buffers containing 10 μM valinomycin, 10 μM nigericin (both Cayman Chemicals, Ann Arbor, MI, USA), 10 μM gramicidin (Sigma-Aldrich), and 0.5 mM ouabain^76^.

### Observation of vacuoles in transmitted light

We used an Olympus IX81 microscope (Center Valley, PA, USA), with a Sensicam QE camera (PCO AG, Germany). Watery vacuoles were recognized either as bright inclusions under a 40x/0.6 phase contrast or by the inversion of contrast at defocusing in bright field (BF) images. Optically denser objects (i.e., with refractive index higher than that of the surrounding medium) have a dark rim at overfocusing (the objective being too far from the object) and a light rim at underfocusing; objects with a lower refractive index behave in the opposite manner.

The dependence of the vacuole appearance on the position of the focal plane is related to the phase delay of light transmitted through an object; at small refraction angles, this relationship is expressed by the transport-of-intensity equation (TIE)^77–79^. The TIE code we used originally^80^ runs on MatLab (MathWorks, Natick, MA, USA); later, we switched to a different code modified as a Fiji plugin^81^. In either case, two BF images were required; they were vertically separated by 1 μm (at approximately ± 0.5 μm of the best focus; the distances were not scaled for refractive index differences) and collected with a 60x/1.42 oil-immersion objective through a 485/10 bandpass filter (Omega Optical, Brattleboro, VT, USA).

### Fluorescence staining

Cells were stained with 1–10 μM Nile Red, 50 nM LysoTracker Red DND-99, 5 μM calcium green (all from Thermo Fisher Scientific), 2 μM DRAQ9 (gift from Roy Edward, BioStatus, UK), 5 μM acridine orange (Immunochemistry Technologies, Davis, CA), or 20 μM BCECF-AM (TEFLabs, Austin, TX, USA). Most fluorescence images were collected on a widefield IX81 or on laser scanning confocal microscopes Fluoview 1000 or Fluoview 3000 (Olympus, Center Valley, PA, USA) using either 60x/1.42 oil-immersion, 60x/1.2 water-immersion, or dry 40x/0.6 phase contrast objective.

For immunofluorescence staining, cells were fixed with 4% formaldehyde. The primary antibodies were monoclonal anti-human LAMP-1 (Biolegend, San Diego, CA, USA), monoclonal rat anti-mouse LAMP-1 (BD Pharmingen, Heidelberg, Germany), or rabbit anti-LC3b (Abcam, Waltham, MA, USA) reactive against human or mouse antigens; they were applied at 1 μg/ml. The secondary antibodies were donkey anti-mouse Alexa 488 and anti-rabbit Alexa 555 or Alexa488-conjugated goat anti-rat (Thermo Fisher Scientific).

### Live-cell imaging

NIH 3T3 cells, C28/I2 cells and C28/I2^LRRC8A−/−^ cells were plated in 35 mm live-cell imaging-suitable petri dishes (ibidi GmbH, Gräfelfing, Germany) using standard culture conditions (37 °C, 5% CO_2_, 95% relative humidity). Live-cell imaging was performed on a NIKON Biostation IQ (Nikon Instruments Inc., Melville, NY, USA). Images were taken at intervals of three minutes for up to 72 h. Videos were analyzed using Nikon NIS Viewer and ImageJ software (NIH).

### Statistical analysis

To assess the effects of various treatments on vacuolation, the following parameters were determined: the percent of cells with at least one clearly visible vacuole (V1), the percent of cells with at least five vacuoles (V5), and the percent of cells with at least one vacuole larger than 2 µm in diameter (V2µm). The numbers are reported as mean ± standard deviation.

## Supplementary Information

Supplementary Video 1.

Supplementary Video 2.

Supplementary Video 3.

Supplementary Video 4.

Supplementary Information.

## Data Availability

All relevant data generated or analyzed during this study are included in this published article and its Supplementary Information files.
