# Supplementary material for: Delayed vacuolation in mammalian cells caused by hypotonicity and ion loss
Source: Sci Rep. 2024 Nov 26;14:29354. doi: 10.1038/s41598-024-79815-z (PMC11599563; doi:10.1038/s41598-024-79815-z)
Supplement: Supplementary file 5 — Supplementary Information. [file 41598_2024_79815_MOESM5_ESM.pdf]

Supplementary Information for

Delayed vacuolation in mammalian cells caused by hypotonicity and ion loss

Emily Zook, Yingzhou Edward Pan, Anna Wipplinger, Hubert H. Kerschbaum, Robert J. Clements, Markus Ritter, Tobias Stauber*, Michael A. Model*

* Corresponding authors: tobias.stauber@medicalschool-hamburg.de (T.S.); mmodel@kent.edu (M.A.M.)

This PDF file includes:

- Supplementary Figures S1 to S11
- Supplementary video legends

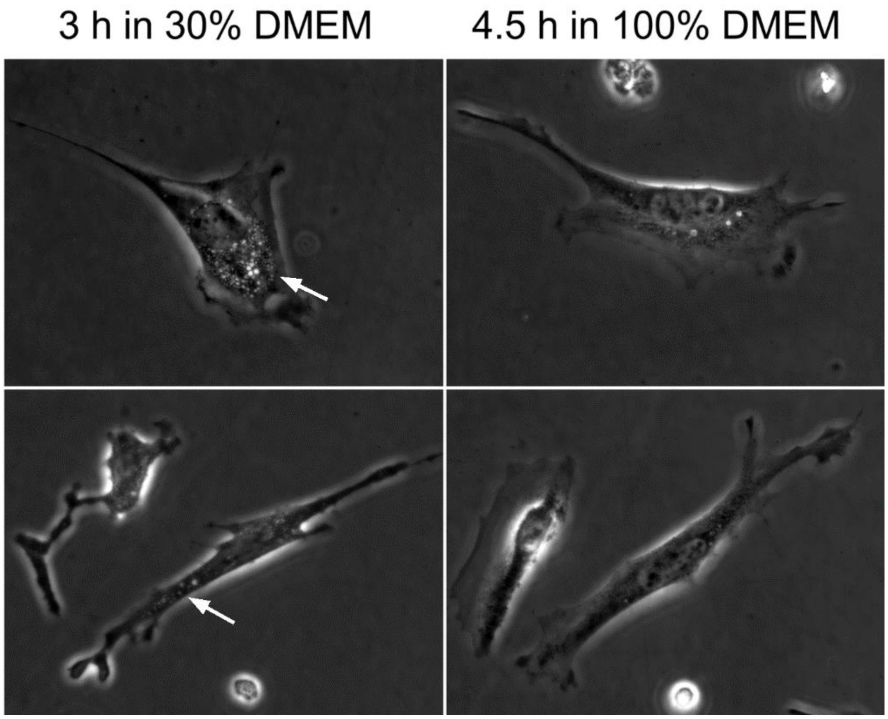

Figure S1. Reversibility of the LateVacs. 3T3 cells were grown in plastic culture dishes and imaged with a phase contrast objective. The first set of images was acquired after 3 h in hypotonic medium; next, the medium was changed to normal DMEM. The second set of images was taken 4.5 hours later. Most vacuoles have disappeared by that time.

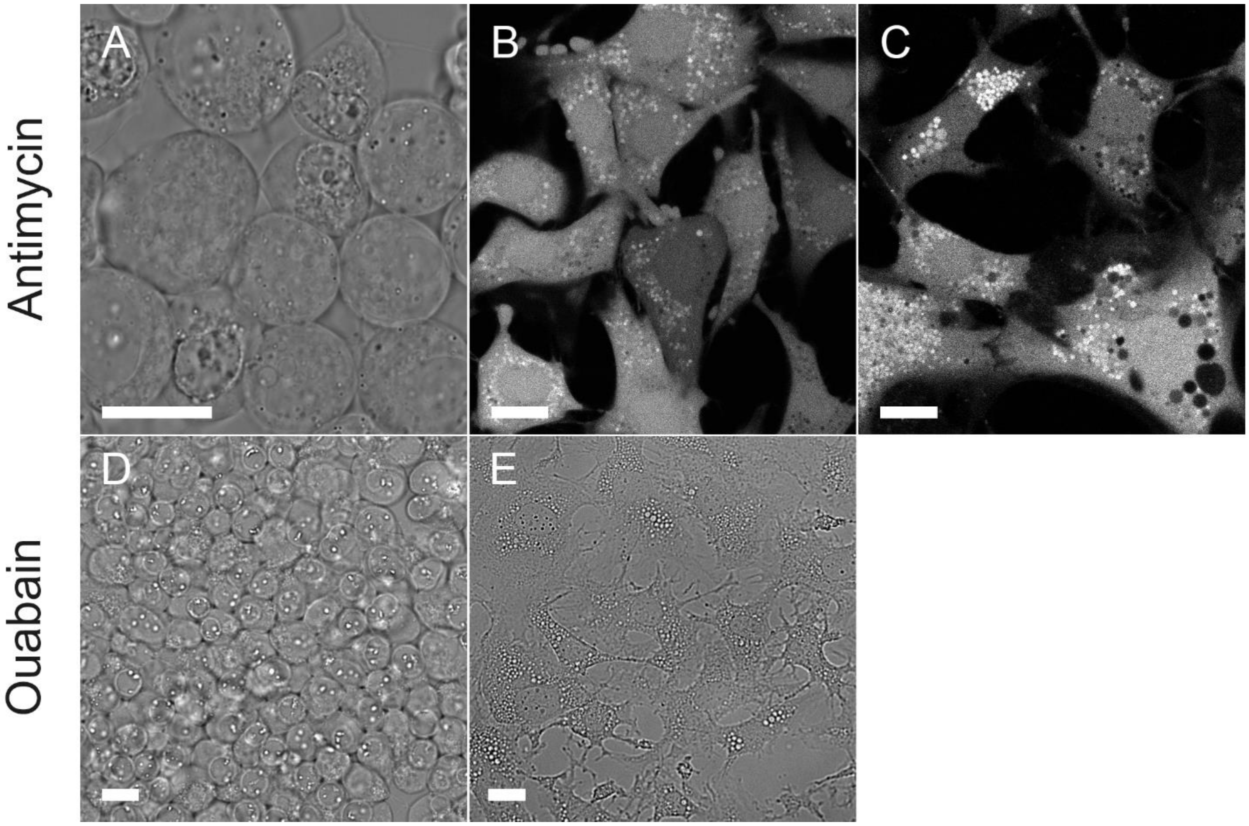

Figure S2. Cell swelling prevents vacuolation. (A) HeLa cells incubated in a hypotonic solution (50% DMEM) in the presence of 16 mM antimycin remain swollen without vacuoles discernable in bright field even under high magnification. (B, C) To verify that their absence was not due to reduced contrast (when surrounded by dilute cytosol), we imaged similarly treated cells on a confocal microscope. Cells were stained with calcein AM before observation. No large vacuoles were visible in antimycin-treated cells (B) but they were prominent in cells incubated without antimycin (C). The small vesicles that accumulate calcein may be precursors of the LateVacs, but the lack of known specificity of calcein makes their identification uncertain. In view of the other results, they are likely to be lysosomes. (D, E). Similar results were obtained when 0.5 mM ouabain was present in 30% DMEM. Ouabain prevented RVD in HeLa but not in 3T3 cells. Accordingly, HeLa remained swollen after 3 hours without visible vacuoles (D) but 3T3 cells became filled with numerous vacuoles (E). Scale bars, 20 μm.

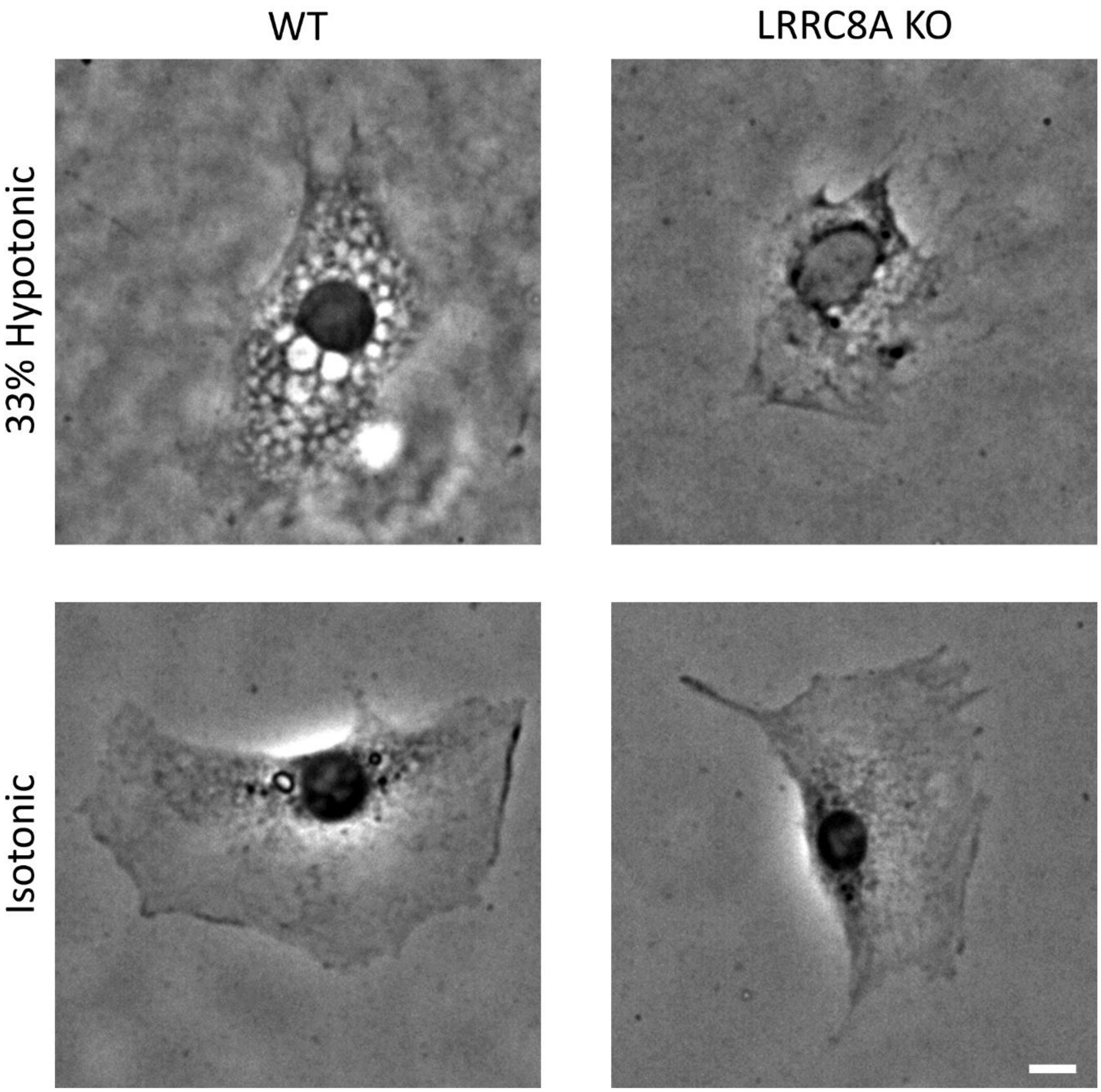

Figure S3. No vacuolation in LRRC8A knockout (KO) 3T3 cells. Phase contrast images of 3T3 cells, either wildtype (WT) or depleted of LRRC8A (KO) after 6 hours in isotonic full DMEM or 33% DMEM. Scale bar, 10 µm.

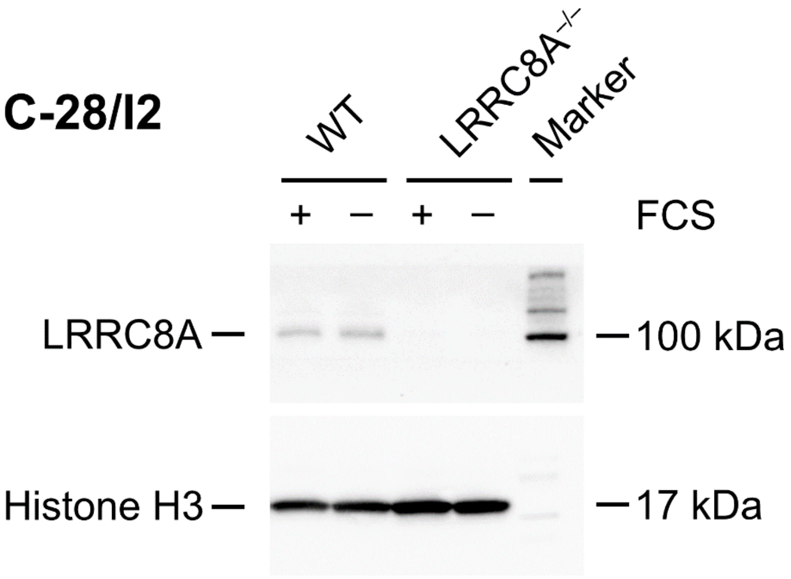

Figure S4. Confirmation of LRRC8A depletion in *LRRC8A*$^{-/-}$ C28/I2 cells. Protein levels of LRRC8A in wildtype (WT) and *LRRC8A*$^{-/-}$ C28/I2 cells grown in the presence or absence of FCS were tested by Western blotting. Histone H3 served as loading control.

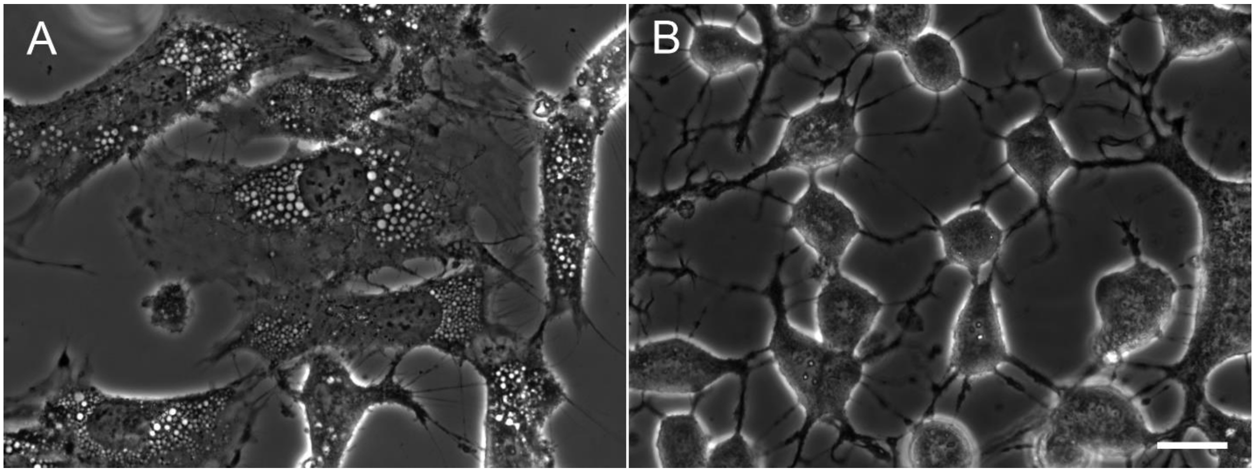

Figure S5. Prevention of vacuolation by chloride channel inhibitor NPPB. 3T3 cells were kept for 4 h in 33% DMEM in the absence of inhibitor (A) or in the presence of 0.5 mM NPPB (B). The vacuole prevalence numbers were V1 = 85%, V5 = 81%, V2mm = 44% without NPPB (n = 41) and near zero (V1 = 4.7%, V5 = 0%, V2mm = 1.3%, n = 233) with NPPB. Similar effect was observed in gluconate buffer: V1 = 36%, V2 = 5%, V5mm = 5% (n = 22). Thus, NPPB at 0.5 mM completely abolished hypotonicity-induced vacuoles. Scale bar, 25 µm.

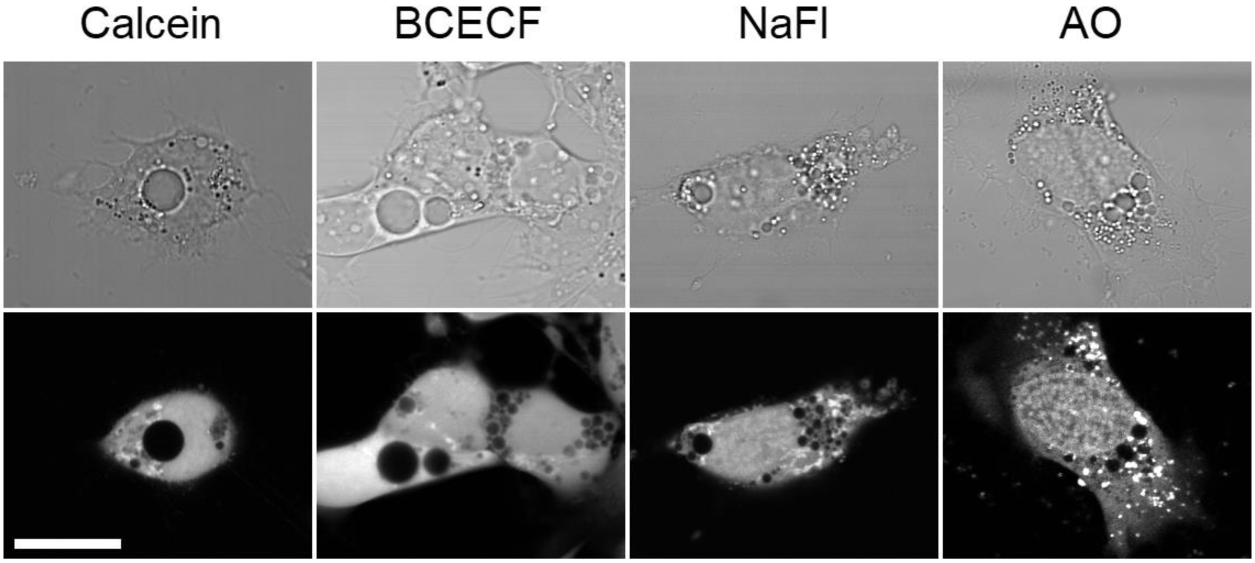

Figure S6. Exclusion of fluorescent dyes calcein AM, BCECF AM, sodium fluorescein (NaFI), and acridine orange (AO) from the LateVacs. Transmission images are shown in the upper row and confocal fluorescence in the lower panel. Fluorescein enters the cells through volume regulated channels (VRAC) that temporarily open in hypotonic solutions[1]. BCECF was used to stain 3T3 cells, and the other dyes were applied to HeLa. Scale bar: 20 μm.

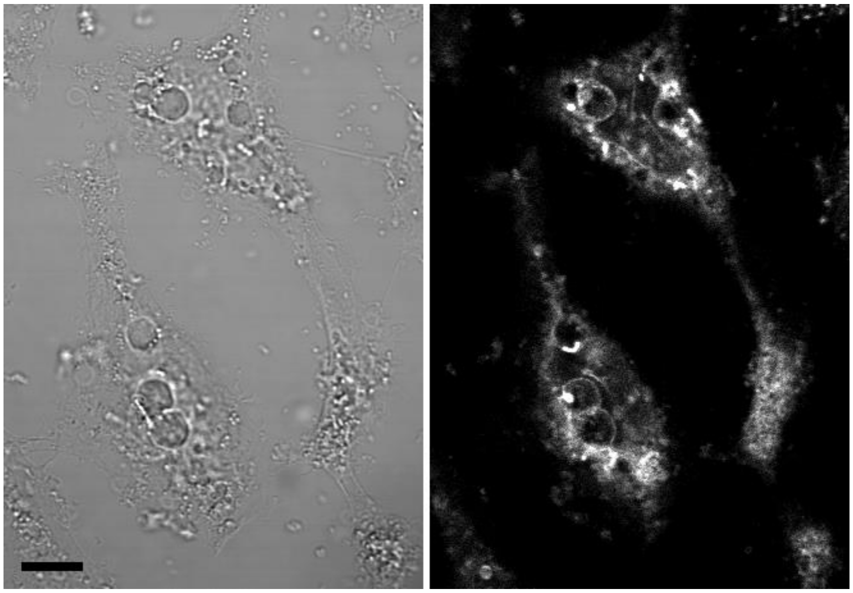

Figure S7. Staining of hypotonically treated HeLa cells with DiBAC$_4$(3). Scale bar, 10 μm.

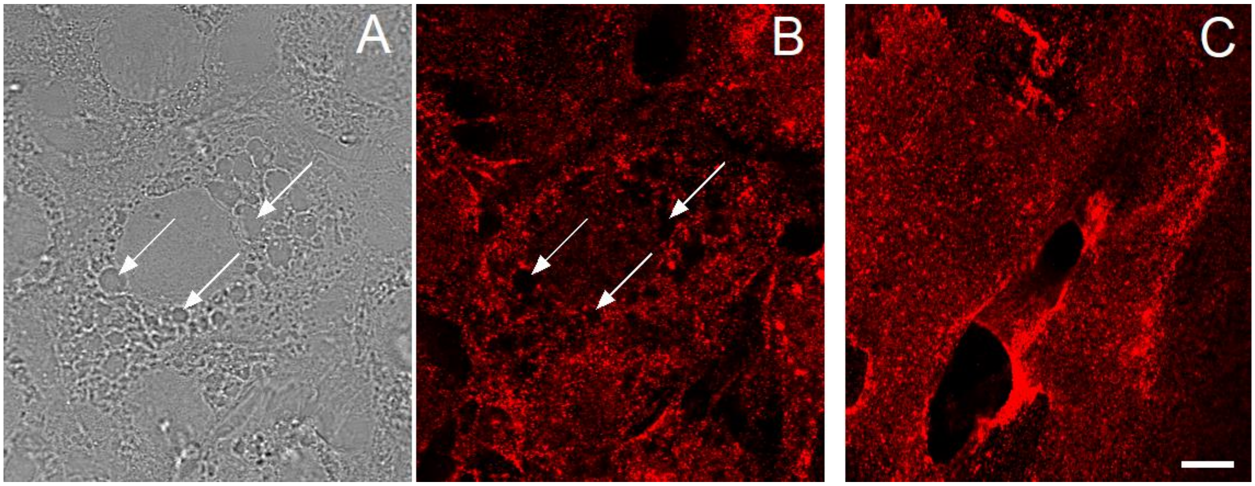

Figure S8. Test for the presence of the autophagy marker LC3 on vacuoles. (A) Brightfield transmission image of hypotonically stimulated 3T3; (B) Anti-LC3 staining; arrows point to vacuoles. No accumulation of LC3 on vacuolar membranes is noticeable. (C) Anti-LC3 staining of untreated cells. All images were obtained on a confocal microscope. Scale bar, 10 µm.

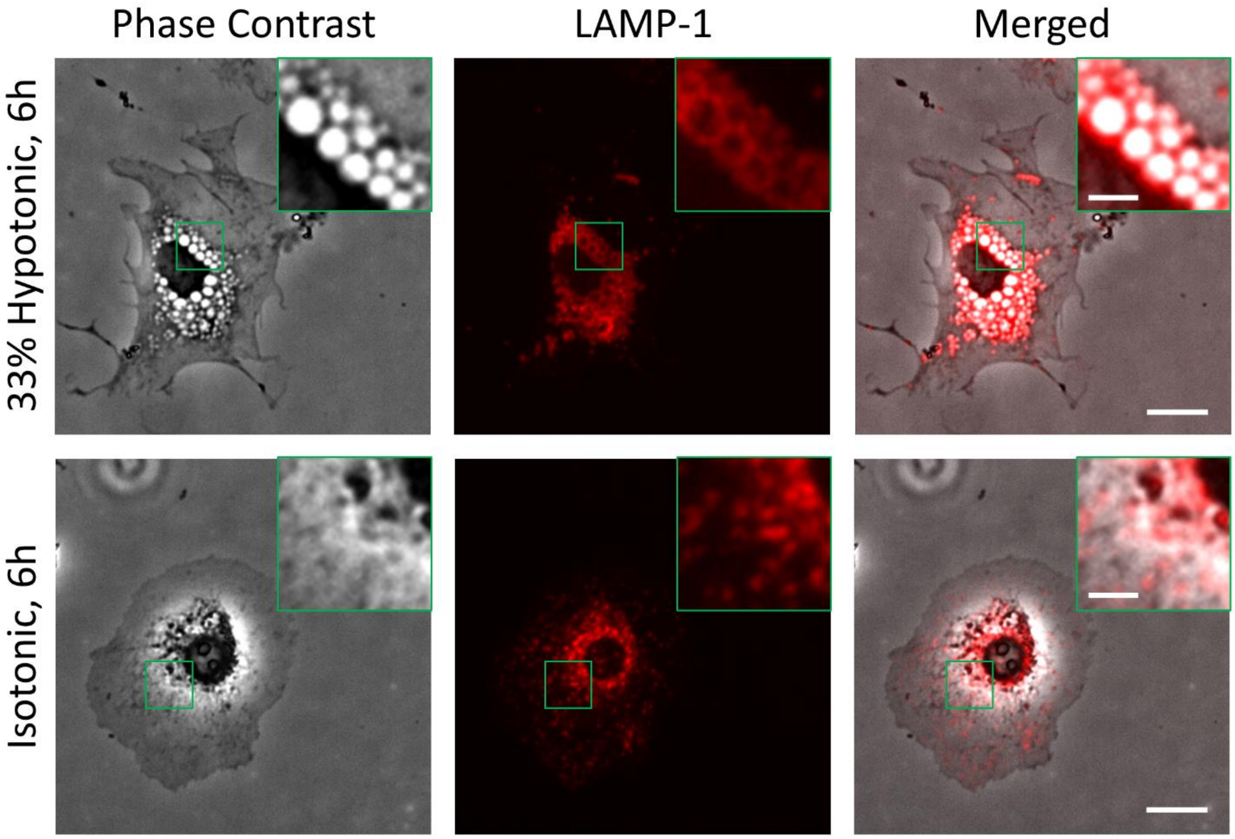

Figure S9. LAMP-1 staining of 3T3 cells after 6 h in 33% DMEM or isotonic medium. Phase contrast, fluorescence, and an overlay are shown. The top right corners show magnified areas containing the stain. Scale bars: 20 µm (main image) or 5 µm (inserts).

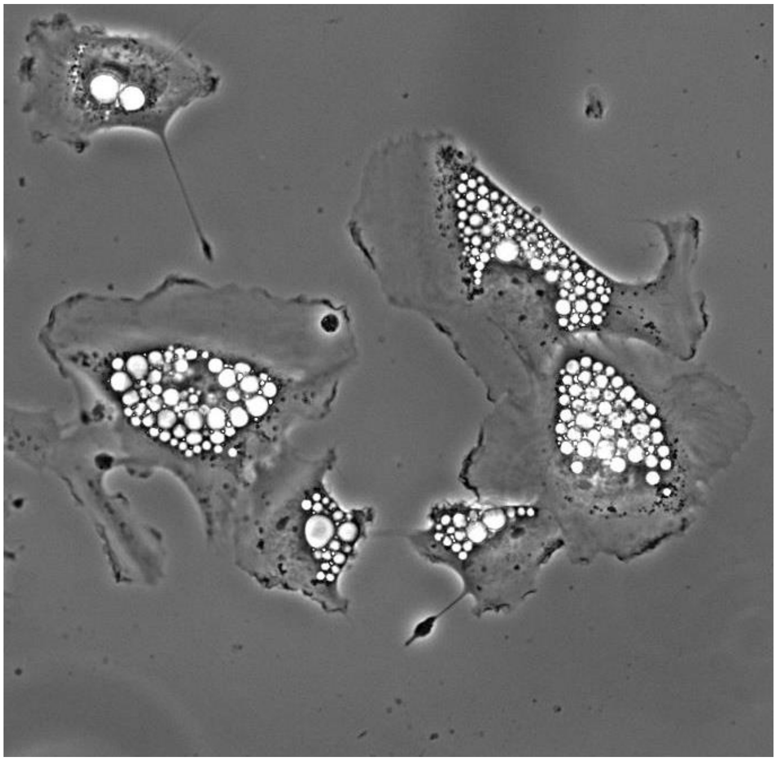

Figure S10. Vacuoles in S28i2 chondrocytes exposed to hypotonic solution.

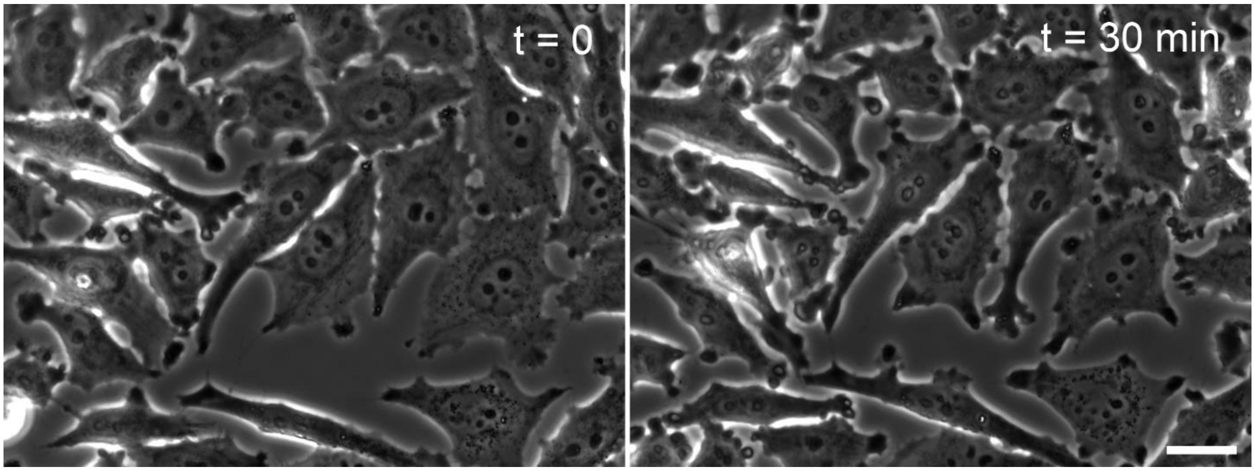

Figure S11. Phase contrast images of HeLa in normal DMEM medium (t = 0) and after 30 min in 50% DMEM. No vacuoles were visible at this time. Scale bar, 25 μm.

Supplementary video legends

Video S1 (C28i_ normotonic condition)
Live-cell imaging of human C28/I2 chondrocytes in normotonic condition. The video starts after 20 minutes, corresponding to minute 0001 of the time indicated in the video, and has a duration of 1300 minutes. The interruption of the video from minute 78 to 84 is due to manual focus readjustment. Characteristically, these chondrocytes were motile, showed ruffling at the leading edge, including the formation of macropinosomes, as well as normotonic cell shrinkage and swelling associated with mitosis. Some chondrocytes showed a single, huge, perinuclear vacuole.

Video S2 (C28i_hypotonic condition)
Live-cell imaging of human C28/I2 chondrocytes exposed to 33% hypotonic medium (1:3 dilution with sterile deionized water). The video starts 20 minutes after hypotonic exposure, corresponding to minute 0001 of the time indicated in the video, and has a duration of 1362 minutes. The interruption of the video from minute 46 to 52 is due to manual focus readjustment. In hypotonic conditions, chondrocytes were immotile, showed intense ruffling at leading edges, increase in vacuoles without detectable pinocytosis, and an increase in cell size.

Video S3 (C28i_LRRC8A_ normotonic condition)
Live-cell imaging of human C28/I2 LRRC8A knockout chondrocytes (which lack functional VRAC), without intervention (*LRRC8A*$^{-/-}$ control). The video starts after 20 minutes, corresponding to minute 0001 of the time indicated in the video, and has a duration of 1357 minutes. The interruptions of the video from minute 131 to 137 and 262 to 271 are due to manual focus readjustment. Chondrocytes were motile, showed intense ruffling at their leading edge, including macropinocytosis, as well as normotonic cell shrinkage and swelling associated with mitosis.

Video S4 (C28i2_LRRC8A_ko_ hypotonic condition)
Live-cell imaging of human C28/I2 chondrocytes exposed to 33% hypotonic medium (1:3 dilution with sterile deionized water). The video starts 20 minutes after hypotonic exposure, corresponding to minute 0001 of the time indicated in the video, and has a duration of 4250 minutes. The interruption of the video from minute 38 to 46 is due to manual focus readjustment. LRRC8A KO chondrocytes showed intense ruffling at leading edge, shrinkage and swelling associated mitosis. These cells did not develop vacuoles.

[1]Model, M. A., Hassani Nia, F., Zook, E., Hollembeak, J. E. & Stauber, T. Uptake of fluorescein upon osmotic cell swelling is dependent on the volume-regulated anion channel VRAC/LRRC8. *P. P. Ex. Med.* **1**, 3-14, doi:10.33594/000000533 (2022).
